# Comparing the Efficacy of Two School-Based Approaches of Neurocognitive Training for Enhancing Executive Functions

**DOI:** 10.3390/children9101501

**Published:** 2022-09-30

**Authors:** Han Jiang, Stuart J. Johnstone, Jinjin Lu

**Affiliations:** 1 School of Special Education, Zhejiang Normal University, Hangzhou 311231, China; 2 School of Psychology, University of Wollongong, Wollongong, NSW 2522, Australia; 3 Department of Educational Studies, Xi’an Jiaotong-Liverpool University, Suzhou 215123, China

**Keywords:** neurocognitive training, teacher positive feedback, inhibitory control, working memory, task switching, ecological validity

## Abstract

Neurocognitive training has been shown to improve a range of cognitive/executive functions and behavior in children. Delivering this training in a school context may enhance its ecological validity and improve training outcomes. The current study examined the efficacy of two school-based neurocognitive training approaches for enhancing executive functions (EF) in typically developing children: neurocognitive training with no teacher positive feedback (NCT), and neurocognitive training with teacher positive feedback (NCT-TPF). Using a randomized control design, 45 children were randomly allocated to the no-training control, NCT, or NCT-TPF group and completed pre- and post-training assessments on the core executive function (EF) domains of inhibitory control, working memory, and task switching. Teachers’ subjective acceptance of the two training protocols was investigated to explore potential ecological validity. The two training groups completed six sessions of training in a kindergarten over a 3-week period. The results showed significant post-training improvements in an untrained inhibitory control task for both training groups when compared with the control group. Different effects were found for each group for the untrained task switching task. While reduced reaction time (RT) in correct Color and Shape trials at Time 2 were reported for the NCT-TPF group, there was no difference compared to the control group for the NCT group. The NCT group showed increased RT in Switch trials but reduced Shape errors compared to controls at Time 2, while these effects were not significant for the NCT-TPF group. An unexpected outcome was that children in both training conditions did not show a significant improvement in an untrained working memory task. Teachers’ subjective acceptance consistently supported including positive feedback as part of NCT. While further research is needed, these results support use of neurocognitive training and/or neurocognitive training with teacher positive feedback for typically developing children in a school context.

## 1. Introduction

Cognitive neuroscience has been increasingly applied to improve cognitive development for children, especially for children with neurodevelopmental disorders (e.g., Attention Deficit Hyperactivity Disorder, ADHD; or Autism Spectrum Disorder, ASD). Common characteristics of these disorders are impairments in learning processes or EFs, which in turn affect learning or socializing [1]. It is common that, while symptoms may have manifested in early childhood, assessment and diagnosis are undertaken at school age. Advantages of applying cognitive neuroscience approaches as a solution are apparent. As non-pharmacological treatments, these approaches avoid the potential side effects of some medications [2,3]. Meanwhile, targeting cognitive/executive functions via measurement and/or manipulation of brain activity has reported better outcomes as compared with psychosocial treatments such as behavioral interventions [4].

Neurocognitive training combines cognitive training (CT) and neurofeedback training (NF) and aims to improve cognitive processes and psychological state control/modulation abilities [5]. CT aims to improve particular cognitive/executive functions (e.g., working memory, inhibitory control) through continuous practice in purpose-designed tasks. These tasks involve performance feedback linked to varied difficulty levels to promote engagement and learning. NF aims to promote awareness and increase self-regulation of brain electrical activity, measured via the electroencephalogram (EEG). Typical goals of neurofeedback training for children with ADHD involve enhancing higher frequency brain activity (e.g., alpha and beta), as well as inhibiting lower frequency activity, e.g., delta and theta; see a review by [6]. Previous studies have reported positive outcomes of CT or NF in reducing the core symptoms of neurodevelopmental disorders such as ADHD [7,8], as well as improving psychosocial outcomes [9]. These approaches can also facilitate the learning process in young children with typical development [10,11].

The form of neurocognitive training examined in the current study was developed to provide a novel and engaging non-pharmacological approach to symptom reduction for children with ADHD [12]. ADHD is a neurodevelopmental disorder with high prevalence which manifests as age-inappropriate and persistent patterns of inattention and/or hyperactivity-impulsivity [1]. While these symptoms may begin in early childhood, assessment and diagnosis commonly occurs at school age when their impact on academic and social functional becomes apparent. Although not listed as symptoms [1], negative social outcomes such as academic underachievement and poor social/peer relationships are common is children with ADHD [13]. The theory underpinning neurocognitive training is the Cognitive Energetic Model (CEM) [14,15]. According to the CEM, ADHD is caused by a state-regulation dysfunction that prohibits efficient engagement of cognitive processes. When functioning effectively, these cognitive processes work together to provide a foundation for an individual’s effective engagement with information in their external world.

Each session of neurocognitive training aims to “exercise” cognitive/executive functions (working memory, inhibitory control) via CT and promote self-regulation of state factors (attention, relaxation) via NF. It is thought that continued practice in these areas boosts the dynamic interplay between them, as theorized by CEM. Our previous studies have reported the effects of NCT in reducing ADHD symptoms and problem behaviors [16], and improving academic on-task behavior and assignment completion [17,18]. These studies also reported good social acceptance by parents/caregivers.

In the past decade, cognitive training research has paid increasing attention to whether improvements in executive functioning transfer to real life performances/outcomes, i.e., show a far transfer effect. Chacko and colleagues proposed that “next generation” neurocognitive training would provide “the cortical foundation” that enables children with neurocognitive impairments to achieve behavioral, academic, and social success in their day-to-day lives by undertaking supportive and skill-based approaches [19]. After all, only if it can help our children to achieve development (preferably perceptible) or improvement (such as academic achievement), can its social value be widely reflected. An emerging research interest is ecological validity and implementation of cognitive training which aims to improve academic achievement [20]. A simplified description of ecological validity, according to Singer [21], is an intervention “delivered by ordinary people under ordinary conditions”. There are at least three components required to realize ecological validity: firstly, an intervention is delivered in a real-life setting, e.g., the school [11], rather than in a setting with controlled factors, e.g., the laboratory [22]. Secondly, it can be delivered and valued by typical agents (e.g., teachers), rather than researchers or developers [18]. Thirdly, it benefits children in coping with difficulties in real-life settings (e.g., mathematics problem-solving), rather than trained and untrained cognitive tasks [23]. 

Following the principle of “delivered by ordinary people under ordinary conditions” [21], we propose that a classroom teacher is an appropriate agent of delivering neurocognitive training in school, for three reasons: Firstly, classroom teachers are very familiar with their students. This may promote training fluency, especially at the early/orientation stage. Secondly, teachers can structure an appropriate training schedule that can be incorporated into the school and classroom routine. Thirdly, teachers are able to play an active role during the training as they know how to instruct and motivate their students. In our preliminary case study [18], a primary school teacher was trained to support the delivery of neurocognitive training completed a 25-session training for two children with diagnosed ADHD (one inattentive subtype, one combined subtype) in an after-school service. We trained the teacher to provide positive feedback to the participating children in each training session. Positive feedback is a simple instructional strategy that has been shown to have a positive influence on student performance [24], including those with ADHD [25].

In the current study, we were interested in the influence of teacher positive feedback (TPF) on training outcomes. TPF was used after one or two neurocognitive training tasks to aid the participating children by: (1) acknowledging student performance during a task, (2) providing advise on how to perform better at a particular task, (3) encouraging the student to attempt a task at a higher level of difficulty, or (4) provide answers to the student’s questions. Further, we were interested to examine training outcomes in typically developed children in a short training span. Previous research has reported immediate changes in cognitive functions (e.g., working memory, attention) after CT [26] or NF [27]. This study adopted a randomized control design that involved three groups. EF and other outcome measures were compared between a group of children who received no training (Control), NCT with no TPF (NCT), and NCT with TPF (NCT-TPF). It was predicted that the NCT-TPF group would show larger beneficial outcomes than the NCT group when each was compared to the control group.

## 2. Materials and Methods

### 2.1. Participants

A total of 45 children recruited from a public kindergarten in Hangzhou (China) participated in this study. These children all had similar socio-economic backgrounds. Written consent was obtained from the parents of all participating children. Children were excluded if they suffered from any chronic disease or clinically diagnosed psychiatric or psychological problem. Each child was randomly allocated to either the control, NCT, or NCT-TPF group. The overall mean age of participants was 6.01 years, with age and sex-ratio for each group shown in Table 1.

### 2.2. Materials 

#### 2.2.1. ADHD Symptoms Scale 

A Chinese version of the SNAP-IV rating scale was used to characterize each participant’s general behavior as rated by their parents [28]. Forty items were used covering DSM-V items for ADHD and ODD, the Conners Index Questionnaire [29], and the IOWA Conners Rating Scale [30]. The Chinese version of the SNAP-IV has been shown to be both reliable and valid (Gau et al., 2008), while the IOWA Conners Rating Scale has shown good internal consistency and test–retest reliability [31]. The items are rated on a 4-point scale from (0) ‘not at all’ to (3) ‘very much’. Average rating-per-item (ARI) were calculated for the DSM-V inattention, hyperactivity/impulsivity, and oppositional defiant domains, and the IOWA aggression/defiance resulting in four SNAP-IV subscale scores that range from 0 to 3. 

#### 2.2.2. Inhibitory Control Task 

Based on earlier adaptive Go-Nogo tasks [32,33], this task required children to press the mouse button on “Go” trials (e.g., pictures of food) and withhold any response on “Nogo” trials (e.g., pictures of anything else). The task design develops a pre-potent tendency to respond as 80% of trials are Go trials, thus requiring participants to inhibit this response on Nogo trials. Forty-two stimuli were delivered, divided evenly over three blocks (with a 30–40 s break in between). Stimulus presentation order was pseudo-random—a block never began with a Nogo trial; no more than two successive Nogo trials. Each trial consisted of an animated stimulus (e.g., a cloche lifting to reveal a food or non-food item) being presented, separated by a 1000 ms inter-stimulus interval. In block 1, the stimulus was presented for 800 ms (level 3), representing the response window. Stimulus presentation durations at other levels were: Level 1, 1600 ms; Level 2, 1200 ms; Level 4, 600 ms; Level 5, 400 ms. If an error was made (i.e., no response to a Go trial, or response to a Nogo trial), the next block was presented at the lower level, or if no errors were made the next block was presented at the higher level. The inhibitory control task, switching task and working memory task (see below) used in this study have been described previously in the study that developed a screening and diagnostic tool for children with ADHD [34].

Each block began with on-screen instructions (“Please click on Food only. Try to be fast and accurate”) with block 1 starting after a practice block of 10 stimuli (80% Go) with subsequent clarification of instructions if required. Derived measures were Go accuracy (%), Nogo accuracy (%), Go RT (ms), and Nogo RT (ms).

#### 2.2.3. Switching Task 

This task required children to sort objects (i.e., red circle, red square, blue circle, blue square) by a sorting dimension (i.e., color or shape) into one of two locations (identified by a blue circle or a red square), and was based on the task reported by Howard and Melhuish [35]. A practice block of 10 trials with sorting on one dimension (i.e., color or shape) was followed by a color sorting block of 15 trials, with approximately 50% blue and red objects. The second block involved a simple rule switch, with sorting of 15 objects according to shape, with approximately 50% circle and square shaped objects. The final “switch” block required the flexible use of both rules based on the border of the object (i.e., sorting was by shape if the object had a black border, and by color if the object had no black border). Within each block, particular color–shape combinations were never presented more than twice in a row.

Each block began with on-screen instructions about the sorting rule (e.g., “Put objects in the box according to their colour”), with blocks separated by a short 3–40 s break and reiteration of instructions. Derived measures were Color errors (sum), Shape errors (sum), Switch errors (sum), Correct color RT (ms), Correct shape RT (ms), and Correct switch RT (ms).

#### 2.2.4. Working Memory Task 

This task required children to search behind a number of doors (range: 2–6) by clicking on them with the mouse, with the aim of locating a person behind each door and was based on a task reported by Morris et al. [36]. The task rules were: (1) do not open doors that have already been opened and did not contain a person, and (2) do not open doors behind which a person has already been found. After initial random allocation to a door, when a person was found another person was then hidden behind another door (never a door behind which a person had been previously found) until a person had been found behind each door—at which point the block would end. One practice block was presented with 4 doors, before three blocks with block 1 containing 4 doors (Level 3). Door numbers at other levels were: Level 1, 2 doors; Level 2, 3 doors; Level 4, 5 doors; Level 5, 6 doors. If an error was made the next block was presented at the lower level, or if no errors were made the next block was presented at the higher level. 

Each block began with presentation or reiteration of the task instructions (i.e., “Find a person behind each door to win the game. Don’t look where you’ve already found a person, or looked before”), with blocks separated by a short 30–40 s break. Measures derived were Search RT (ms) and Task Accuracy (%).

Each participant also completed several questionnaires that are not reported here, including a modified version of the Children’s Report of Sleep Patterns [37], a modified version of the Children’s Report of Sleep Patterns—Sleepiness Scale [38], a modified version of the Basic Psychological Needs Scale [39,40], and the EQ-5D Youth [41,42]. The participant’s parents also completed a modified Basic Psychological Needs Scale [39,40], and the Child Self-Regulation and Behaviour Questionnaire [35]—these measures are not reported here.

#### 2.2.5. Subjective Acceptance of the Training Agents 

After completion of the training, all of the training agents were asked to write a reflective comment about their subjective acceptance of the training protocols. Specifically, they were asked two questions: (1) “Which training protocol (NCT or NCT-TPF) would you choose for your future students?”, and (2) “Can you provide some reasons for this choice?”. The answers were typed anonymously, printed, and then sealed in envelopes before submitting to the researchers. This procedure prevented the researchers identifying the author by his/her name or handwriting. The reflections were interpreted following the spiral process that consisted of data managing, reading and memoing, describing, classifying, and interpreting [43]. Themes were generated and ordered according to the number of supporting agents. 

### 2.3. Procedure

#### 2.3.1. Pre- and Post-Training Assessment 

All the children completed the inhibitory control task, switching task, and working memory task one day before and one day after the training regime. Parents and homeroom teachers completed the Chinese version of the SNAP-IV within one week before and after the training schedule. The homeroom teachers were not training agents who delivered NCT or NCT-TPF to participating children. Parents were blind to the group assignment. The homeroom teachers were blind to training group assignment.

#### 2.3.2. Training Protocols

##### Software

Focus Pocus is a themed software application owned by the University of Wollongong. The participant adopts the role of “a wizard in training”, tasked with practicing skills such as broomstick racing or levitation to become a skillful wizard. Each training session is made up of fourteen games: four inhibitory control (e.g., Goblin Bashing), four working memory (e.g., Find the spell), and six NF (e.g., Transformation) games presented in a random order. The NF games involve EEG input measured as described below, with gaming elements controlled by dynamic change in live EEG input (e.g., the speed of the participant’s wizard avatar in a broomstick race again computer-controlled wizards is dependent on their current level of EEG alpha power). For the six NF games, two are driven by attention (linked primarily to EEG beta power), two by relaxation (linked primarily to EEG alpha power), and two an averaged attention and relaxation index (termed “Zen”). The inhibitory control games require a tap/press response to frequently presented “Go” stimuli and the withholding of response to infrequent “Nogo” stimuli. The working memory games involved holding information in memory with subsequent recall to complete an action. During a NF game, the player needs to self-regulate an appropriate level of attention, relaxation, or Zen for one minute. All games involve adaptive difficulty levels, in that an increment of 5% follows successful completion the previous level and a decrement of 5% follows an incomplete previous level. Completing a training session independently takes 15 to 20 min.

##### Hardware

A dry-sensor EEG recording device (Mindwave^TM^, Neurosky, San Jose, CA, USA) was used to control gameplay during NF games, and quantify attention level during the inhibitory control and working memory games. The device is a headband that contains microchips, embedded firmware (ThinkGear, Neurosky, San Jose, CA, USA), a 10 mm active electrode, and an ear-clip reference ground electrode. EEG was record continuously from site Fp1 at 256 Hz, with on-board conversion of the raw signal from the time- to the frequency-domain via a fast Fourier transformation to calculate EEG power in the delta, theta, alpha, and beta frequency bands for more information, see [5]. These measures are presented as a value between 0 and 100, for providing robust and universal feedback about ongoing EEG activity in a form understood by teachers and children. These values were sent to computer or iOS device wirelessly via Bluetooth. The device has been shown to be reliable and valid [44,45].

##### Training Protocol for the NCT Group 

The NCT group completed the training sessions with very limited feedback from teachers. While the child was completing the training sessions, a teacher sat beside them to monitor the process. No feedback was provided to the child.

##### Training Protocol for the NCT-TPF Group 

The NCT-TPF group completed the training sessions with positive feedback from the teachers (i.e., TPF). Previously, positive feedback has been highly recommended as a powerful strategy during instructions and task-related events [24,46]. Compared with reinforcement and punishment, providing high informational feedback (e.g., cues, or specific information about doing a task) and expressing positive affect (e.g., acknowledgement, encouragement) have higher effects in mediating students to stay on the right track (e.g., attention, behavior, emotion, and strategies) in pursuing further success [46]. This may facilitate a student’s performance during cognitive training as that student may encounter difficulties (e.g., meeting an advanced game level, seeking a new strategy for completing a task) and thus generate negative feelings (e.g., be afraid of hardship, doubting ability). During the NCT-TPF condition, the teacher sat beside the child while they were completing the games. The TPF was expected to promote the child’s self-evaluation, problem solving, positive affect, and motivation during training. Table 2 provides information on the types of TPF and examples. Instances of TPF were limited to 5 to 10 times per training session.

Both the NCT and NCT-TPF groups completed six training sessions in a three-week period (2 sessions per week). All children learned how to use the software during a face-to-face session. A practicum session was applied before the formal training to ensure children’s mastery. The training was conducted in the resource classrooms or conference rooms of the participating kindergarten. The resource classrooms are primarily for providing academic aid or consulting services for children with special needs. Both rooms were quiet and isolated from teaching classrooms and interruptions. All sessions were conducted between 9:00 a.m. and 11:00 a.m. from Monday to Friday.

##### Training Agents

Ten late-stage undergraduates majoring in Special Education were recruited as the training agents. These undergraduates had passed their teacher qualification requirements and had registered licenses in teaching. A rigorous training program was undertaken by all agents to ensure practice consistency. Initially, the agents were trained in the background and techniques associated with children with ADHD and NCT (10 h). Secondly, the agents were trained in the spiral process. Subsequent training involved rounds of role-playing of the teacher role in the NCT and NCT-TPF protocols, with feedback from the trainer and fellow agents until the protocol had been mastered (10 h). 

### 2.4. Statistical Analyses 

To examine Group differences for age and SNAP-IV subscale scores, univariate ANOVAs with a between-subjects factor of Group (Control, NCT, NCT-TPF) were utilised. To assess the effect of training on the EF task performance measures, univariate ANOVAs considered Group (Control, NCT, NCT-TPF) effects at Time 2 with Time 1 as a covariate. Planned contrasts compared the Control and NCT groups, and Control and NCT-TPF groups.

## 3. Results

Sex-ratio did not differ significantly between groups (χ^2^ (2) = 0.729, *p* = 0.70). Mean age was significantly higher in the NCT-TPF than control group (F = 5.281, *p* = 0.009, $\eta_{p}^{2}=$ 0.205) and was therefore used as a covariate in all subsequent analyses, with adjusted effects reported if relevant. See Table 1.

None of the four SNAP-IV subscale scores differed significantly between the groups—see Table 1. The sample’s DSM-V subscale scores were similar to those reported for the general population of 6-year old children in Taiwan (i.e., Inattentive 0.81, Hyperactivity/Impulsivity 0.77, Oppositional Defiant 0.74; GAU et al., 2008) and in the USA, i.e., Inattentive 0.62, Hyperactivity/Impulsivity 0.75, Oppositional Defiant 0.54 [47] when rated by parents. Further, the IOWA subscale scores were similar to those reported for Canadian children aged 5–12 years, i.e., Oppositional Defiant 0.62 [31].

### 3.1. Inhibitory Control Task 

**Figure 1:**
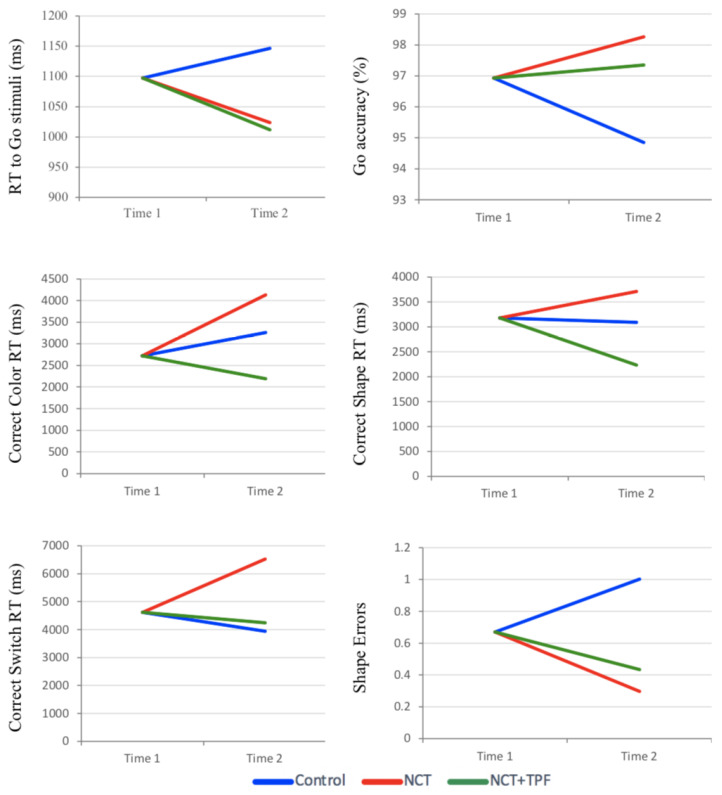
Significant difference at Time 2 with Time 1 as a covariate for RT to Go Stimuli, Go Accuracy, Correct Color RT, Correct Shape RT, Correct Switch RT, and Shape Errors.

A significant Group main effect (F = 7.85, *p* = 0.001, η^2^ = 0.277) and planned contrasts revealed that Go RT was significantly reduced in the NCT group compared to the Control group (1023.7 vs. 1146.4 ms, *p* = 0.002) and in the NCT-TPF group compared to the Control group (1011.8 vs. 1146.4 ms, *p* = 0.001) at Time 2, with Time 1 as a covariate (Figure 1).

### 3.2. Working Memory Task 

The Group main effect for both Search RT and Task Accuracy at Time 2, with Time 1 as a covariate, was not significant.

### 3.3. Switching Task 

A significant Group main effect (F = 5.041, *p* = 0.011, η^2^ = 0.197) and planned contrasts revealed that Correct color RT was reduced in the NCT-TPF group compared to the Control group (2192.6 vs. 3260.7 ms, *p* = 0.086), with no difference between the NCT and Control groups (4131.2 vs. 3260.7 ms) at Time 2, with Time 1 as a covariate (Figure 1). A significant Group main effect (F = 5.103, *p* = 0.010, η^2^ = 0.199) and planned contrasts revealed that Correct Shape RT was reduced in the NCT-TPF group compared to the Control group (2232.6 vs. 3090.6 ms, *p* = 0.068), with no difference between the NCT and Control groups (3710.9 vs. 3090.6 ms) at Time 2, with Time 1 as a covariate. A significant Group main effect (F = 3.369, *p* = 0.044, η^2^ = 0.141) and planned contrasts revealed that Correct Switch RT was increased in the NCT group compared to the Control group (6522.2 vs. 3940.3 ms, *p* = 0.022), with no difference between the NCT-TPF and Control groups (4243.6 vs. 3940.3 ms) at Time 2, with Time 1 as a covariate. A near-significant Group main effect (F = 2.605, *p* = 0.086, η^2^ = 0.113) and planned contrasts revealed that Shape Errors were reduced in the NCT group compared to the Control group (0.297 vs. 1.002, *p* = 0.038), with no difference between the NCT-TPF and Control groups (0.434 vs. 1.002) at Time 2, with Time 1 as a covariate. 

### 3.4. Training Agents’ Subjective Acceptance

To the question “Which training protocol (the NCT or NCT-TPF) will you choose for your future students?” all training agents (n = 10) selected the NCT-TPF. Six themes were generated to support their choice of NCT-TPF (see Table 3).

## 4. Discussion

The current study was the first randomized control trail to examine the efficacy of two school-based neurocognitive training protocols for enhancing executive functions in typically developing children. Three tasks covering the core EF domains of inhibitory control, working memory, and task switching were used to examine training outcomes from the gamified training tasks which aimed to exercise and improve inhibitory control, working memory, and psychological state regulation processes. Note that when the training tasks and tasks assessing training outcomes overlapped, they involved similar processes but shared few surface features. This study represents an extension of previous approaches [16,17] by using trained pre-service teachers to provide either no feedback (the NCT condition) or intensive positive feedback (the NCT-TPF condition) to the child during the training sessions. The current study also investigated the teacher’s subjective acceptance of the two training protocols as part of an exploration of potential ecological validity.

Children in the NCT and NCT-TPF groups showed improvements in an untrained inhibitory control task when compared with the control group. This effect on inhibitory control processes was not reported in our previous studies for children with clinically significant ADHD or subclinical ADHD [5,16]. It is unclear if this difference is due to the current sample consisting of typically developing and not clinical/sub-clinical participants, or perhaps the age difference between studies; 6 years in the current and ranging from 7 to 12 years in previous studies. From a developmental perspective, it is thought that cognitive training in early childhood is likely to produce more widespread effects [10]. Nevertheless, the findings of the current study add to evidence that supports the application of cognitive neuroscience approaches which promote exercise (and perhaps plasticity) of cognitive processes in early childhood in the form of programmed training protocols [10].

An unexpected outcome was that children in both training conditions did not show a significant improvement in an untrained working memory task. A possible reason for this outcome is the shorter training duration, and hence reduce training trail exposures, in this compared to previous studies. Klingberg [48] emphasized that training to promote plasticity of working memory processes is optimized by intensive and extended training schedules. Similarly, as noted by Sprenger et al. [49] studies that reported efficacies of working memory training involved participants many hours of training (e.g., 20 h) or training over several weeks (4 to 6 weeks). Some studies, e.g., [27] that report poor outcomes of training in cognitive functions (included working memory) have discussed insufficient training intensities as a contributing factor. Future studies of short duration training protocols would likely benefit from increasing the per session training trial exposures in the working memory domain.

There were different effects for the NCT and NCT-TPF groups when looking at the influence of training on task switching processes. While reduced RT in correct Color and Shape trials at Time 2 were reported for the NCT-TPF group, indicative of improvement in response speed during these trial types, there was no difference compared to controls for the NCT group. Additionally, the NCT group showed increased RT in Switch trials compared to controls at Time 2, indicative of reduced response speed (and perhaps a more conservative response strategy), with no difference found for the NCT-TPF group. Shape errors were reduced in the NCT group at Time 2 compared to controls, and while this effect was not significant for the NCT-TPF group, it should be noted that there was a large reduction in shape errors for this group. It is worthwhile noting that the task switching task was a completely untrained measure of EF in this study, and that task switching is seen as a separate but core component of EFs along with inhibitory control and working memory. While more work is needed to replicate these effects, the results presented here suggest that NCT and NCT-TPF, which primarily targets the processes of inhibitory control, working memory, and psychological state regulation, can positively influence performance in an untrained but related EF domain.

The training agents (i.e., teachers) consistently supported including positive feedback as part of NCT. In the reasons given, themes such as “promoting training effects” and “enhancing mastery” were endorsed by all or most teachers, respectively. These findings speak to ecological validity and have important implications. TPF allowed teachers to identify “gaps” in the computerized training program that could be complemented instantly via their guidance (e.g., the program presents a 0–5 star rating of performance on each game, and assumes the child knows that 5 stars is better than 2, but this can be quickly addressed by the teacher). TPF promoted the belief in teachers that they themselves were capable of assisting to enhance training outcomes. TPF allowed teachers to detect and respond to fluctuations in enjoyment of or engagement with the training, and provide feedback and support to positively influence this—as evidence by several themes (i.e., increasing the child’s self-efficacy, increasing the child’s motivation). This addresses an issue noted in one of our previous studies where we determined that children’s engagement with and enjoyment of the training declined across sessions [16] and that incorporating personal best goals with NCT resulted in a better behavioral outcomes and child engagement [50]. The results of the current study suggest that it is worthwhile for future research to further investigate the effect of teacher positive feedback in optimizing NCT outcomes.

Despite suggestions that CT, or NF, or NCT should be used in school contexts [9,19], understanding how to do this in a way that generates the expected outcomes and does not negatively affecting academic learning will require in-depth investigation. One of the critical factors is “buy-in” or acceptance of the approach by teachers and other stakeholders (e.g., school administrators). Another important factor is coordination of the training with school routines. One study to deliver NF is a school context reported difficulties related to scheduling time for training and unexpected scheduling changes La Marca and O’Connor [51]. Careful planning and discussions with stakeholders may provide a foundation for easier and more effective implementation, as has been well-modeled by the Positive Behavioral Interventions & Supports (PBIS) program, a school-based program for supporting children with social, emotional, and behavioral needs [52].

Results of the current study should be interpreted in light of some limitations. Firstly, the training agents were pre-service teachers who majored in Special Education. Given that they were not employed school-teachers, their opinions might may differ from those who were formally employed in some situations [53]. Future research may consider involving in-service teachers to implement the training. Secondly, although all training agents received a rigorous training program of providing the PTF, it is possible that, on a few occasions, a training agent provided insufficient feedback to the child. The findings of the current study could not rule out the possibilities that insufficient feedback might induce similar effects as the NCT-PTF group resulted. Future research may compare the effects between the NCT-PTF group and the NCT group with neutral feedback (e.g., You completed the current task, please go to the next task.) or the NCT group with positive but irrelevant feedback to the task (e.g., You are a good child.). Thirdly, all of the participating children were recruited from one public kindergarten that is a typical public kindergarten in Hangzhou, China. The findings of the current study might be associated with this particular school context, and might therefore reduce the generalizability of our results. Lastly, the current study measured children’s EFs as an outcome of neurocognitive training. Future research may also measure children’s performance in day-to-day activities to inform ecological validity [54].

## 5. Conclusions

The current study has extended research into the efficacy of neurocognitive training to the school context. It demonstrates that teachers with proper training can take the agent role for delivery of neurocognitive training to typically developing children in kindergarten. The findings provide evidence for efficacy of the neurocognitive training approach for improving inhibitory control and task switching abilities. No significant improvements were found in working memory, and future research should investigate whether this outcome was due to the short training period. From the teachers’ perspective, neurocognitive training can be facilitated by their positive feedback. Overall, the findings of current study support the use of neurocognitive training and/or neurocognitive training with teacher positive feedback for typically developing children in a school context.

## Figures and Tables

**Table 1:** Age, sex, DSM-V, IOWA and Conners scores for each group at pre-training stage.

	Group		
	Control	NCT	NCT-TPF
Age	5.95 (0.31)	5.86 (0.34)	6.23 (0.32)
Male/Female	10/5	8/7	8/7
DSM-V Inattentive	0.68 (0.26)	0.66 (0.58)	0.97 (0.46)
DSM-V Hyperactivity/Impulsivity	0.65 (0.47)	0.70 (0.56)	0.87 (0.39)
DSM-V Oppositional Defiant	0.71 (0.36)	0.58 (0.40)	0.79 (0.42)
IOWA Aggression/Defiance	0.67 (0.38)	0.66 (0.52)	0.72 (0.35)

**Table 2:** Types and examples of teacher feedback during a neurocognitive training session.

Type	Description	Example
1. Performance	Providing specific information about how well the student did on the current task.	You did well in this game because you looked at the picture and checked the color before pressing the mouse.
2. Advice	Providing specific advice on how to improve the student’s performance on a particular task.	Next time, sit back, relax, and think about wanting to fly faster than your competitors (in the “Broomstick Racing” game).
3. Encouragement	Encouraging the student to exert more effort or try a harder level.	You did excellent work in the “Find the Spells” game. I am confident that you will achieve a good score in the next level.
4. Inquiry	Answer student’s questions about the training.	Student: I beat all the goblins but why did I not go up to a higher level? Teacher: Yes, you have done your work correctly. To go to a higher level, you need to be fast and accurate.

Note. This table duplicated from Jiang, H., Johnstone, S. J., Sun, L., and Zhang, D.-W. (2021). Effect of Neurocognitive Training for Children with ADHD at Improving Academic Engagement in Two Learning Settings. Journal of Attention Disorders.

**Table 3:** Themes generated from the training agents’ reflections.

Theme	Number of Supporting Agents (?/10)	Examples from Raw Material
Promoting training effects	10/10	When incorporating TPF in (NCT) training, the child can do the training better and better. The effect will be better. I will add positive feedback. By doing this the child can know where he is doing well, so he can do better and better, and the effect will be better.
Increasing the child’s self-efficacy	9/10	Sometimes children will be afraid of difficulties. Positive feedback can increase children’s confidence.
Increasing the child’s motivation	8/10	I think it’s also some encouragement for children. Otherwise, for the first time and the second time, the children will be interested in doing it. However, when the children take more sessions, they may not want to do it without receiving feedback.
Enhancing mastery	7/10	It can provide guidance on methods for children and help them practice improving their ability of attention, memory, and self-control.
Supporting positive teacher-student relationships	3/10	Combined with positive feedback will have a good interaction with children, otherwise it seems very “indifferent” and awkward (during the training). I think the process will be more natural and the atmosphere will be better if teachers’ positive feedback is incorporated.
Getting to know the updated situation of the child in the training	2/10	I can instantly understand the specific situation of students.

## Data Availability

The data presented in this study are available on request from the corresponding author. The data are not publicly available due to ethical requirements.
